# Beneficial effects of tri-lithium pyrroloquinoline quinonein on behaviors and pathology in a mouse model of Alzheimer’s disease

**DOI:** 10.1186/1750-1326-7-S1-O14

**Published:** 2012-02-07

**Authors:** Lei Zhao, Neng Gong, Xiaojing Sun, Zhe Yu, Qi Fang, Na Zhao, Xiaoli Pan, Tianle Xu, Chunjiu Zhong

**Affiliations:** 1 Department of Neurology, Zhongshan Hospital, Fudan Univeristy, Shanghai 200032, China; 2 Institute of Neuroscience, State Key Laboratory of Neuroscience, Shanghai Institutes for Biological Sciences, Chinese Academy of Sciences, China

## 

Alzheimer’s disease (AD) is a complex disease with characteristic pathological hallmarks of senile plaques and neurofibrillary tangles, the occurrence and development of which are involved in multiple neurodegenerative processes. Conventional AD therapies target only at single disease-causing mechanisms. However, these have been mostly shown to be ineffective in recent clinical trials. Here we tested a novel strategy using a synthetic organic lithium salt, tri-lithium pyrroloquinoline quinonein (Li_3_PQQ), to modulate mulitple pathways involved in AD pathogenesis. The study of acute toxicity with mice showed Li3PQQ with a very low toxicity (Li3PQQ ID50 5g/kg weight vs. LiCl ID50 2.5g/kg weight). We showed that 8 weeks of daily Li_3_PQQ administration in the APP/PS1 mice significantly improved the learning and memory function in the Morris water maze test and facilitated long-term potentiation. Li_3_PQQ significantly reduced the area and numbers of amyloid plaque and phosphorylated tau levels in cortical areas and mechanistically, it increased activities of Aβ-binding alcohol dehydrogenase but decreased activities of glycogen synthase kinase-3 in the transgenic mice. Therefore, Li_3_PQQ exhibits profound beneficial effects on cognitive impairment and pathological alterations in the AD mouse model. Our study demonstrates the effectiveness of a novel therapeutic strategy for AD through targeting at multiple disease-causing mechanisms.

